# Cytokines Induced by *Edwardsiella tarda*: Profile and Role in Antibacterial Immunity

**DOI:** 10.3390/biom11081242

**Published:** 2021-08-19

**Authors:** Huili Li, Boguang Sun, Shuai Jiang, Li Sun

**Affiliations:** 1 CAS Key Laboratory of Experimental Marine Biology, Institute of Oceanology, Center for Ocean Mega-Science, Chinese Academy of Sciences, Qingdao 266071, China; lihuili@qdio.ac.cn (H.L.); sunboguang@qdio.ac.cn (B.S.); sjiang@qdio.ac.cn (S.J.); 2 Laboratory for Marine Biology and Biotechnology, Pilot National Laboratory for Marine Science and Technology (Qingdao), Qingdao 266237, China; 3 College of Earth and Planetary Sciences, University of Chinese Academy of Sciences, Beijing 100049, China

**Keywords:** cytokine, *Edwardsiella tarda*, infection, antibody array, immune response

## Abstract

*Edwardsiella tarda* is a Gram-negative bacterial pathogen with a broad range of hosts, including fish and mammals. In the present study, we used an advanced antibody array technology to identify the expression pattern of cytokines induced by *E. tarda* in a mouse infection model. In total, 31 and 24 differentially expressed cytokines (DECs) were identified in the plasma at 6 h and 24 h post-infection (hpi), respectively. The DECs were markedly enriched in the Gene Ontology (GO) terms associated with cell migration and response to chemokine and in the Kyoto Encyclopedia of Genes and Genomes (KEGG) pathways associated with immunity, diseases, and infection. Ten key DECs, including IL6 and TNF-α, were found to form extensive protein-protein interaction networks. IL6 was demonstrated to inhibit *E. tarda* infection and be required for *E. tarda*-induced inflammatory response. TNF-α also exerted an inhibitory effect on *E. tarda* infection, and knockdown of fish (Japanese flounder) TNF-α promoted *E. tarda* invasion in host cells. Together, the results of this study revealed a comprehensive profile of cytokines induced by *E. tarda*, thus adding new insights into the role of cytokine-associated immunity against bacterial infection and also providing the potential plasma biomarkers of *E. tarda* infection for future studies.

## 1. Introduction

Cytokines are small, soluble proteins produced by certain cells that act largely in a paracrine way to influence the activity of other cells [1]. Cytokine–mediated effects are critical to many biological processes, including inflammation, antimicrobial immunity, and cancer [2,3,4,5]. Cytokines are the most important class of mediators and can amplify and coordinate pro-inflammatory signals that lead to the expression of effector molecules, resulting in the modulation of diverse aspects of innate immunity against infection [6,7]. Currently, the term “cytokine” encompasses many types of proteins, including interleukins, chemokines, and the tumor necrosis factor (TNF) family. Interleukins mediate interactions between immune cells and are able to promote cell proliferation, differentiation, and functional activation [8,9,10,11]. Chemokines induce cell migration and activation by binding to specific G-protein-coupled cell surface receptors on target cells, such as neutrophils, monocytes, and lymphocytes, which play a pivotal role in host immune defense [12,13,14]. TNF–α is a factor with potent pro-inflammatory activity [15]. Members of the TNF family are involved in apoptosis, proliferation, invasion, and the immune response to infection [16,17].

*Edwardsiella tarda* is a Gram-negative bacterium and a pathogen to fish, reptiles, birds, amphibians, and mammals [18,19,20]. Several studies have indicated that *E. tarda* is able to replicate in fish and mammalian phagocytes and resist the killing of serum complements [21,22,23,24,25]. In addition, *E. tarda* has been reported to invade and replicate in epithelial cells such as HeLa, HEp-2, and flounder gill cells (FG-9307) [26,27,28]. The typical clinical signs caused by *E. tarda* infection include bacteremia and bloody colitis, suggesting a systemic spread of *E. tarda* from epithelium to tissues [29]. *E. tarda* infection is known to induce varied immune responses in different hosts. In zebrafish (*Danio rerio*), *E. tarda* infection upregulated the mRNA levels of interleukin (IL)–1β and TNF–α [20]. In Indian major carp (*Labeo rohita*), *E. tarda* challenge upregulated IL–1β, inducible nitric oxide synthase (iNOS), complement component C3, and downregulated TNF–α [30]. In ginbuna crucian carp (*Carassius auratus langsdorfii*), *E. tarda* stimulated the innate immune response as well as the cytotoxic activity of cytotoxic T lymphocytes (CTLs) and increased the number of CD8^+^ cells, which contributed to the elimination of the bacteria from the tissues [31]. In Japanese flounder (*Paralichthys olivaceus*), *E. tarda* elicited a strong response of microRNAs (miRNAs) and their target genes, which in turn promoted/attenuated *E. tarda* invasion [32,33,34,35]. In mice, our previous work showed that living and non-living *E. tarda* induced strikingly different transcriptome profiles in macrophages, especially the genes associated with immunity [36].

Antibody array is a novel technology developed to meet the growing demand for multiplexed protein detection and can be applied to the simultaneous detection of multiple proteins [37]. In this study, in order to gain a new understanding of the role of cytokines in *E. tarda* infection, we employed an antibody array to examine systematically the plasma cytokine profiles of mice infected with *E. tarda* at different time points. We uncovered a large number of cytokines induced by *E. tarda* and investigated the antibacterial effects of some of the cytokines. In addition, we also applied the findings in mice to fish and examined the potential importance of fish cytokine to the defense against *E. tarda* infection.

## 2. Materials and Methods

### 2.1. Animals and Cell Lines

Clinically healthy BALB/c mice (female, 6–8 weeks, and 18 ± 2 g) were purchased from Qingdao Daren Fortune Animal Technology Co., Ltd. (Shandong, China) Before the experiment, mice were acclimatized in the laboratory for one week as reported previously [38]. RAW264.7 cells were purchased from American Tissue Culture Collection (ATCC, Rockville, MD, USA). The cells were cultured at 37 °C in DMEM medium (Invitrogen, Carlsbad, CA, USA) supplemented with 10% fetal bovine serum (FBS) (Gibco, Grand Island, NY, USA), 100 units/mL penicillin, and 100 µg/mL streptomycin (Beyotime, Shanghai, China) in a humidified atmosphere containing 5% CO2. The Japanese flounder cell line FG–9307 [39] was cultured at 24 °C in L–15 medium (Sigma, St Louis, MO, USA) containing 10% FBS, 100 units/mL penicillin, and 100 μg/mL streptomycin. 

### 2.2. In Vivo Infection

The *E. tarda* strain used in this study was isolated from diseased fish [40]. *E. tarda* was inoculated (1:100) into Luria–Bertani (LB) medium (5 mL) and incubated at 28 °C to an OD_600_ of 0.8. The bacteria were collected by centrifugation at 8000× *g* for 2 min at room temperature. The bacterial pellet was washed with PBS and resuspended in PBS to 5 × 10^8^ CFU/mL. In vivo infection was performed as reported previously [38] with a slight adjustment. Briefly, BALB/c mice (described above) were divided randomly into two groups and injected intraperitoneally (i.p.) with 100 µL *E. tarda* suspension or PBS (control). At 6 h, 12 h, and 24 h post-infection (hpi), blood, liver, and spleen were taken aseptically from the mice (3 animals/time point). The tissues were homogenized in PBS, and bacterial numbers in the homogenates were determined by plate count [38]. Blood collected from mice was placed in EDTA–K2 anticoagulant tube (KWS, Shijiazhuang, Hebei, China) and centrifuged at 2000× *g* to separate the plasma.

### 2.3. Antibody Array and Cytokine Determination

The plasma collected above was used to detect cytokine profiles with the mouse cytokine array G1000 (RayBiotech, Norcross, GA, USA), which can simultaneously detect 96 separate cytokines, according to the instructions of the manufacturer. According to the internal positive control provided by RayBiotech, the signal values were read and normalized. Proteins with a fold change ≥1.5 and adjusted *p*-value < 0.05 were considered as significantly and differentially expressed cytokines (DECs).

### 2.4. Functional Enrichment Analysis of the DECs

Gene Ontology (GO) functional enrichment was performed using R/Bioconductor (http://www.bioconductor.org/, accessed on 17 May 2021). Kyoto Encyclopedia of Genes and Genomes (KEGG) pathway analysis was performed using the Kyoto Encyclopedia of Genes and Genomes database (https://www.kegg.jp/kegg/rest/keggapi.html, accessed on 17 May 2021). After multiple test corrections, GO terms and KEGG pathways with adjusted *p* values < 0.05 were considered to be significantly enriched in DECs. Protein-protein interaction (PPI) networks were constructed with the DECs (Table 1) using STRING v10.0 (http://string-db.org/, accessed on 17 May 2021) with default parameters. The STRING database provides a critical assessment and integration of protein-protein interactions, including direct (physical) as well as indirect (functional) associations [41]. In this study, PPI networks were constructed using STRING v10.0 with the minimum required interaction score set at a high confidence level (score: 0.700).

### 2.5. Cellular Infection

RAW264.7 cells were infected with *E. tarda* as described previously [42] with slight modification. Briefly, *E. tarda* was prepared as described above and resuspended in PBS to 1 × 10^8^ CFU/mL. *E. tarda* was added to RAW264.7 cells in a 24-well plate at a multiplicity of infection (MOI) of 5:1. The plate was centrifuged at 400× *g* for 10 min, followed by incubation at 30 °C for 1 h. After incubation, the supernatant of the culture was removed. To kill extracellular *E. tarda*, fresh Opti-MEM (Gibco, Grand Island, NY, USA) containing gentamicin (200 µg/mL) (Solarbio, Beijing, China) was added to the plate, and the plate was incubated at 30 °C for 40 min. The cells were then washed three times with PBS and cultured in Opti-MEM containing 30 µg/mL gentamicin for 0, 2, 4, and 6 h to allow intracellular replication of the bacteria. At each time point, 300 µL 1% Triton X–100 was added to the plate to lyse the cells. The lysate was diluted and plated onto LB agar plates supplemented with 30 µg/mL tetracycline (Solarbio, Beijing, China). The plates were incubated at 28 °C for 24–48 h, and the number of colonies was counted. To examine the effect of cytokines on *E. tarda* infection, RAW 264.7 cells were treated with or without (control) 50 ng/mL recombinant IL6 (rIL6) (BioLegend, San Diego, CA, USA) for 2 h, 200 ng/mL rTNF–α (BioLegend, San Diego, CA, USA), 100 ng/mL rCSF1 (Sino Biological, Beijing, China), or 200 ng/mL rIL12B (Sino Biological, Beijing, China) for 8 h prior to infection, and then infected with *E. tarda* as above. Flounder FG–9307 cells were infected with *E. tarda* as described previously [34] with a slight adjustment. *E. tarda* was added to FG–9307 cells in a 24-well plate at an MOI of 5:1. The plate was centrifuged at 400× *g* for 10 min, followed by incubation at 28 °C for 1 h. After incubation, the supernatant of the culture was removed. To kill extracellular *E. tarda*, fresh L–15 medium containing gentamicin (200 µg/mL) was added to the plate, and the plate was incubated at 28 °C for 40 min. The cells were washed three times with PBS and cultured in L–15 medium containing 30 μg/mL gentamicin for 0, 2, 4, and 6 h. At each time point, the cells were lysed to determine the intracellular bacterial number as above.

### 2.6. Gene Knockdown by RNA Interference

The siRNAs used in this study were synthesized by GenePharma (Shanghai, China). The sequences of the siRNAs are listed in Supplementary Materials Table S1. To interfere with IL6 expression, RAW264.7 cells were transfected with or without (control) IL6si or NCsi for 24 h using Lipofectamine RNAiMAX (Invitrogen, Carlsbad, CA, USA) according to the instructions of the manufacturer. To interfere with PoTNF–α expression, FG–9307 cells were transfected with or without (control) PoTNF–αsi or NCsi as above. Gene knockdown was verified by quantitative real-time PCR (qRT–PCR) as described below and shown in Supplementary Material Figure S1. Infection of the gene knockdown cells with *E. tarda* was performed as described above.

### 2.7. RNA Extraction and Quantitative Real-Time PCR (qRT–PCR)

To determine the expression of IL6 and PoTNF-α during *E. tarda* infection, RAW264.7 cells, and FG–9307 cells were infected with *E. tarda* as described above. To determine the effect of IL6 knockdown on inflammatory cytokine expression during *E. tarda* infection, RAW264.7 cells were pretreated with or without (control) IL6si or NCsi and then infected with *E. tarda* as described above. Total RNA of the infected cells was extracted with RNA–easy isolation reagent (Vazyme, Nanjing, China). The RNA was used for cDNA synthesis with First Strand cDNA Synthesis Kit (ToYoBo, Osaka, Japan) according to the manufacturer’s protocol. qRT–PCR was carried out with Eppendorf Mastercycler epgradient S (Eppendorf, Hamburg, Germany) using TB Green Premix Ex Taq™ II (Takara, Dalian, China). The sequences of the primers used for qRT–PCR are listed in Supplementary Materials Table S2. The expression of each gene was normalized to that of glyceraldehyde–3–phosphate dehydrogenase (GAPDH) (for mouse genes) or β–actin (for flounder genes) [43] and calculated using the comparative threshold cycle method (2^−ΔΔCT^). The assay was performed in triplicate.

### 2.8. Determination of Nitric Oxide (NO) Production

RAW264.7 cells were pretreated with or without (control) IL6si or NCsi and then infected with *E. tarda* as described above. NO production was determined at 2, 4, and 6 hpi using the DAF–FM DA fluorescent probe (Beyotime, Beijing, China) according to the manufacturer’s instructions. The experiment was performed three times.

### 2.9. Statistical Analysis

All experiments were performed three times. Statistical analyses were performed using student’s *t*-tests and one-way analysis of variance (ANOVA) in GraphPad Prism version 6.01 (GraphPad Software Inc., San Diego, CA, USA). The results were considered statistically significant when *p* < 0.05.

## 3. Results

### 3.1. Detection of E. tarda–Induced Cytokines

**Figure 1:**
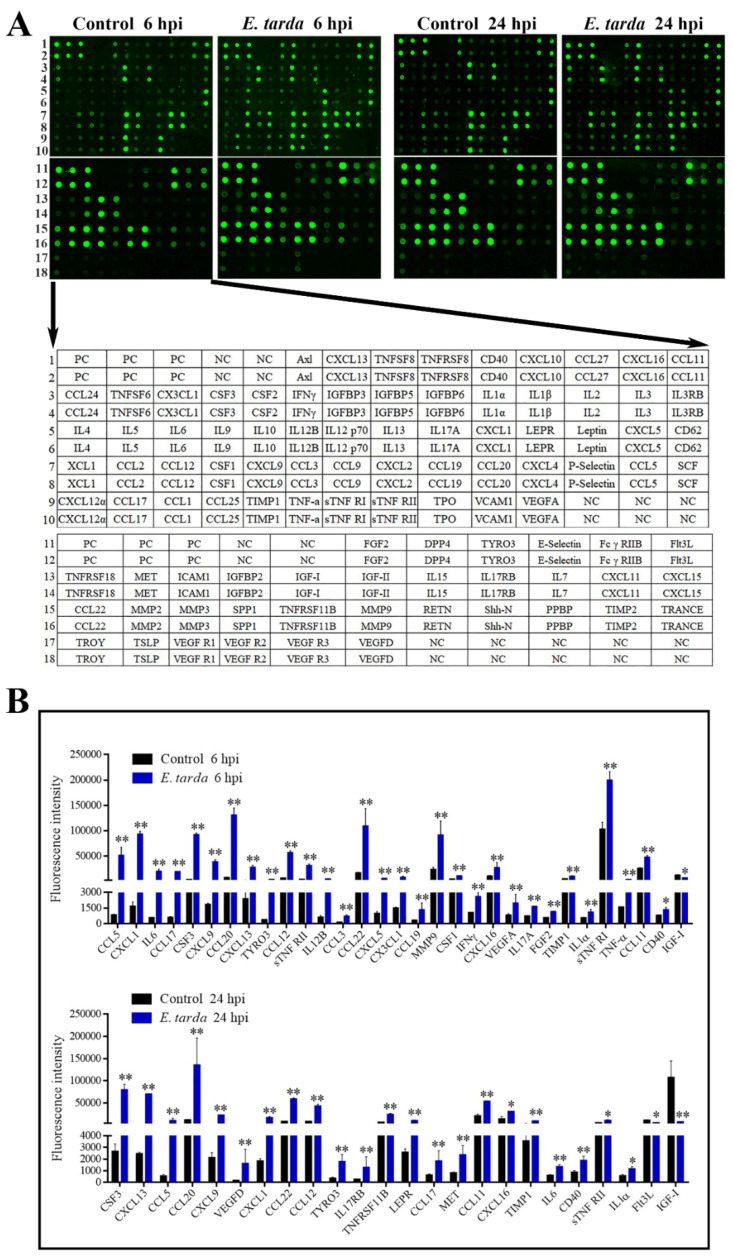
The cytokine expression profiles induced by *Edwardsiella tarda*. (**A**) Mice were infected with or without (control) *E. tarda*, and the expressions of 96 cytokines in the plasma at 6- or 24-hour post-infection (hpi) were detected by an antibody array (upper panel). Each cytokine was assayed in duplicate. The cytokine spots are indicated in the lower panel. PC, positive control; NC, negative control. The data shown are one representative of triplicate experiments. (**B**) Differentially expressed cytokines at 6 hpi (upper) and 24 hpi (lower) are shown in the histogram. Data are the means of triplicate assays and shown as mean ± SD. * *p* < 0.05; ** *p* < 0.01.

Following infection of mice, *E. tarda* disseminated into the liver, spleen, and blood in a time-dependent manner (Supplementary Materials Figure S2). *E. tarda*–induced production of 96 cytokines at 6 and 24 hpi was examined by antibody array (Figure 1A). Cytokines with a fold change ≥ 1.5 and an adjusted *p*-value < 0.05 were considered as differentially expressed cytokines (DECs). The expression levels and fold changes of the DECs are shown in Figure 1B and Table 1, respectively. At 6 hpi, 31 DECs were detected, 30 of which were upregulated. At 24 hpi, 24 DECs were detected, 22 of which were upregulated. Among the DECs, CCL5, CXCL1, IL6, CCL17, colony stimulating factor (CSF) 3, CXCL9, CCL20, CXCL13, TYRO3 protein tyrosine kinase (Tyro3), CCL12, tumor necrosis factor receptor superfamily member 1b (sTNF RII), CCL22, CXCL16, tissue inhibitor of metalloproteinase 1 (TIMP1), IL1–α, CCL11, and CD40 were upregulated at both 6 hpi and 24 hpi; whereas, insulin-like growth factor 1 (IGF–I) was downregulated at both 6 hpi and 24 hpi.

### 3.2. GO and KEGG Analysis of the DECs

**Figure 2:**
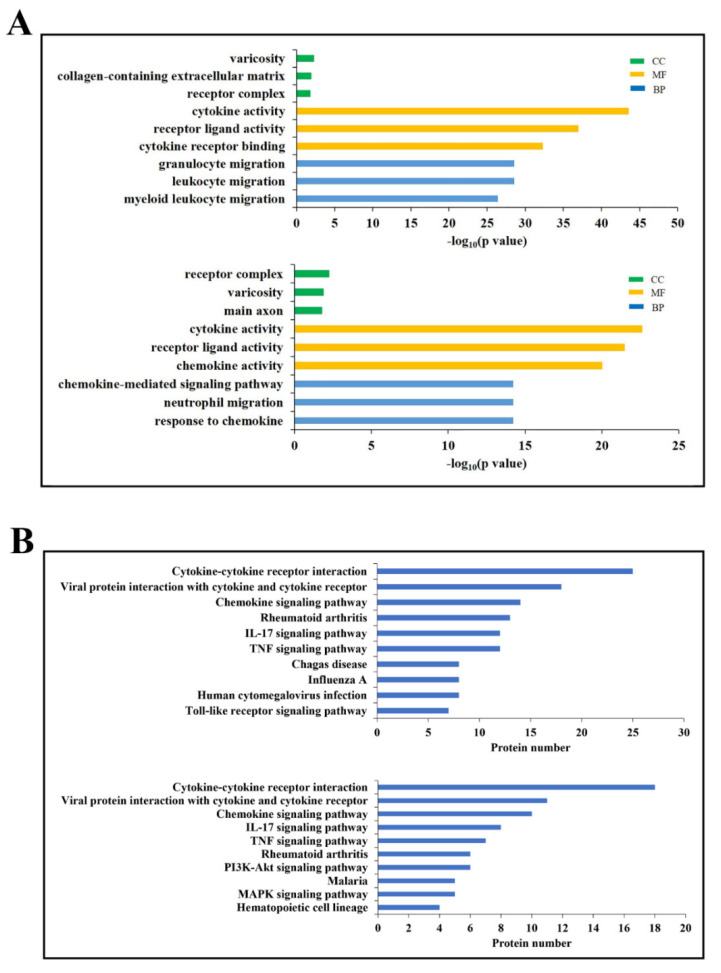
GO and KEGG enrichment of differentially expressed cytokines (DECs). (**A**) DECs of the top three significantly enriched GO terms at 6 (upper) and 24 (lower) hours post-infection (hpi). CC: Cellular Component, MF: Molecular Function, BP: Biological Process. (**B**) DECs of the top 10 significantly enriched KEGG pathways at 6 (upper) and 24 (lower) hpi.

DECs enriched in the top three GO functional terms are shown in Figure 2A. In the category of biological process, cytokines associated with granulocyte migration, leukocyte migration, and myeloid leukocyte migration were highly represented at 6 hpi, while cytokines of chemokine–mediated signaling pathway, neutrophil migration, and response to chemokine were highly represented at 24 hpi. In the category of molecular function, cytokine activity and receptor-ligand activity were highly represented at both 6 and 24 hpi; cytokine receptor binding and chemokine activity were highly enriched at 6 and 24 hpi, respectively. In the category of cellular components, receptor complex and varicosity were represented at both 6 and 24 hpi; collagen-containing extracellular matrix and main axon were represented at 6 and 24 hpi, respectively.

DECs of the top 10 most abundant KEGG pathways are shown in Figure 2B. Six of the 10 pathways were detected at both 6 and 24 hpi, including cytokine-cytokine receptor interaction, viral protein interaction with cytokine and cytokine receptor, chemokine signaling pathway, Rheumatoid arthritis, IL–17 signaling pathway, and TNF signaling pathway. Of the other pathways, chagas disease, influenza A, human cytomegalovirus infection, and toll-like receptor signaling pathway were highly represented at 6 hpi; pl3K–Akt signaling pathway, malaria, MAPK signaling pathway, and hematopoietic cell lineage were highly represented at 24 hpi.

### 3.3. The Interaction Networks of the DECs

**Figure 3:**
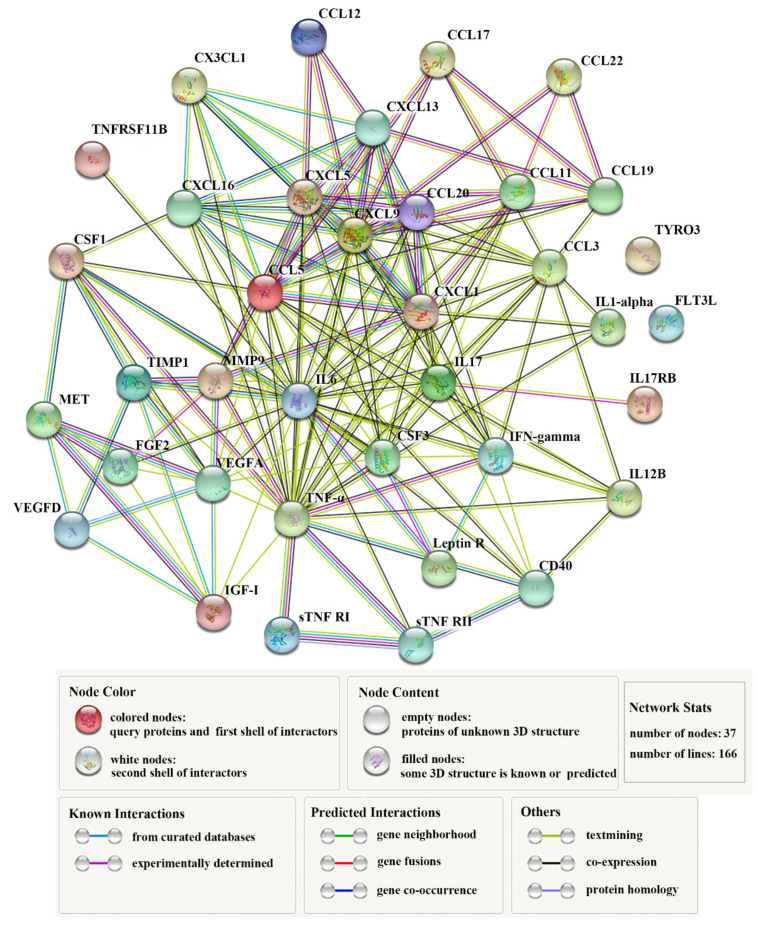
The interaction networks of differentially expressed cytokines (DECs). The networks formed by 37 DECs are shown. Nodes in the networks represent proteins; lines indicate association between the linked DECs.

Thirty-seven DECs (Table 1) constituted complicated interaction networks (Figure 3), in which multiple interactive relationships were formed among the DECs. Table 2 lists the top 10 key DECs with the highest numbers (≥ 13) of protein-protein interactions. Of these cytokines, IL6 displayed the highest number (26) of interactions. Next to IL6 was TNF–α, which interacted with 24 DECs. Other highly interactive DECs included CCL5, CXCL9, IL17, CXCL1, CXCL5, CCL3, CSF3, and VEGFA, with interaction numbers ranging between 13 and 19 (Table 2).

### 3.4. Effects of the DECs on E. tarda Infection

**Figure 4:**
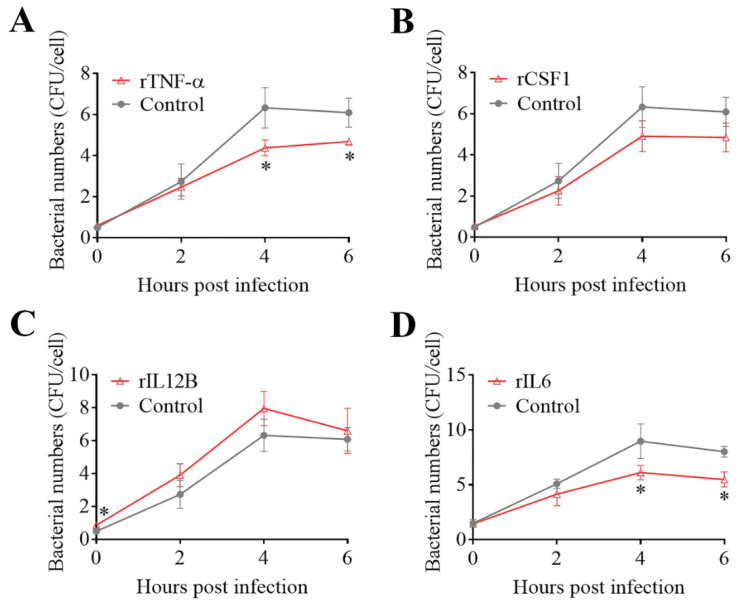
Effects of DECs on *Edwardsiella tarda* infection. RAW264.7 cells were pretreated with rTNF–α (**A**), rCSF1 (**B**), rIL12B (**C**), or rIL6 (**D**) and infected with *E. tarda* for 1 h, and the extracellular bacteria were killed by antibiotic treatment. The cells were then incubated for various times. After incubation, the intracellular bacterial number (shown as colony forming units, CFU) was determined. Values are the means of triplicate experiments and shown as mean ± SD. * *p* < 0.05.

**Figure 5:**
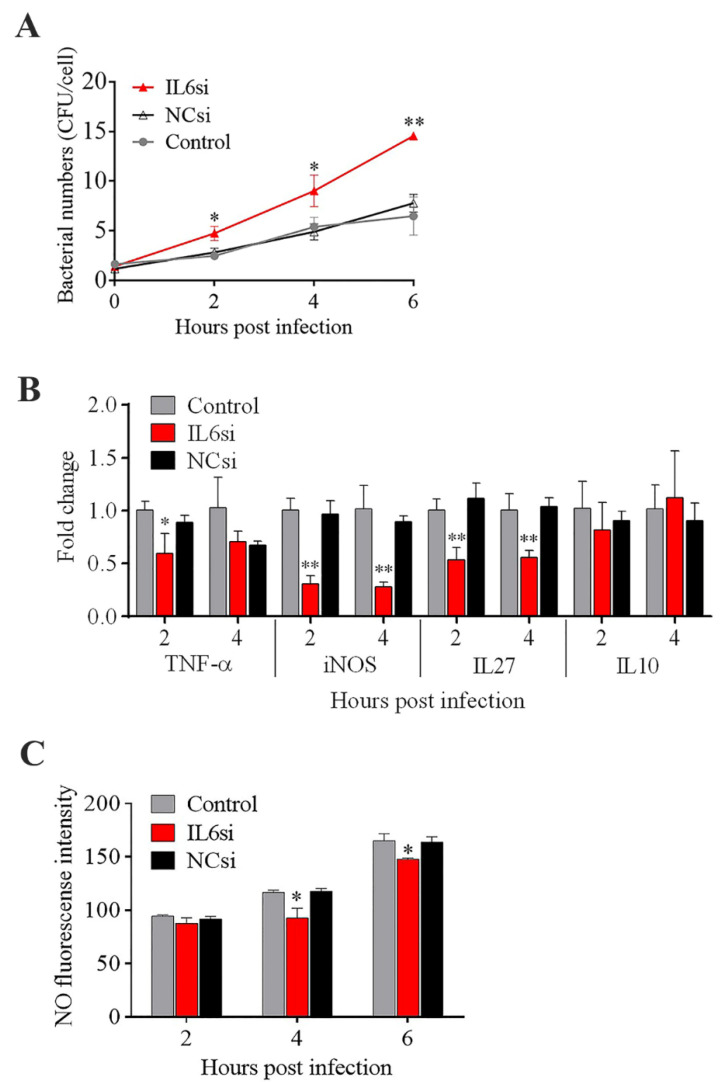
Effect of IL6 knockdown on *Edwardsiella tarda* infection and inflammatory cytokine expression. RAW264.7 cells treated with or without (control) IL6si (a siRNA targeting IL6) or NCsi (negative control siRNA) were infected with *E. tarda* for 1 h, and the extracellular bacteria were killed by antibiotic treatment. The cells were then incubated for different hours. After incubation, the intracellular bacterial number (**A**), the expression of inflammatory genes (**B**), and nitric oxide (NO) production (**C**) were determined. Values are the means of triplicate experiments and shown as mean ± SD. * *p* < 0.05; ** *p* < 0.01.

The effects of some of the key DECs on *E. tarda* infections in mouse macrophages (RAW264.7 cells) were examined. Pretreatment of the cells with rTNF–α and rIL6 significantly decreased the intracellular replication of *E. tarda* at 4 and 6 hpi, whereas pretreatment with rCSF1 had no significant effect on *E. tarda* infection (Figure 4A,B,D). The intracellular bacterial load in the cells treated with rIL12B was significantly higher than that in the control cells at 0 hpi but not at later time points (Figure 4C), suggesting that rIL12B likely affected the processes of bacterial attachment and invasion. During *E. tarda* infection, IL6 expression was found to increase significantly (Supplementary Materials Figure S3). Interference with IL6 expression markedly enhanced the intracellular infection of *E. tarda* (Figure 5A). IL6 knockdown significantly reduced the expression of TNF–α, iNOS, and IL27 during *E. tarda* infection, but had no effect on the expression of IL10 (Figure 5B). IL6 knockdown also caused a significant inhibition of NO, but not ROS, production (Figure 5C and data not shown).

### 3.5. Effect of Japanese Flounder TNF–α (PoTNF–α) on E. tarda Infection

**Figure 6:**
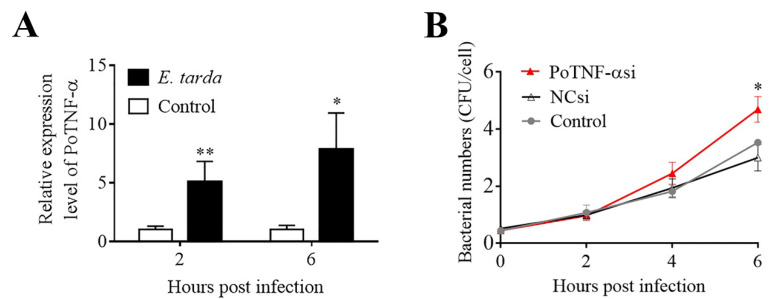
Effect of Japanese flounder TNF–α (PoTNF–α) knockdown on *Edwardsiella tarda* infection. (**A**) Flounder FG–9307 cells were infected with *E. tarda* for 1 h, and the extracellular bacteria were killed by antibiotic treatment. The cells were then incubated for 2 and 6 h, and PoTNF–α expression was determined by qRT–PCR. (**B**) FG–9307 cells were pre-treated with or without (control) PoTNF–αsi (a siRNA targeting PoTNF–α) or NCsi (negative control siRNA) and infected with *E. tarda* for 1 h. The extracellular bacteria were killed as above. The cells were then incubated for different hours, and the intracellular bacterial number was determined. Values are the means of triplicate experiments and shown as mean ± SD. * *p* < 0.05; ** *p* < 0.01.

Since, as shown above, TNF–α is a key DEC during *E. tarda* infection, we examined its involvement in *E. tarda* infection in Japanese flounder, an aquaculture fish highly susceptible to *E. tarda*. We found that the expression of PoTNF–α in flounder cells was significantly upregulated by *E. tarda* at 2 and 6 hpi (Figure 6A). In flounder cells with PoTNF–α knockdown, the intracellular numbers of *E. tarda* were comparable to that of the control cells at 2 and 4 hpi but were significantly higher than that of the control cells at 6 hpi (Figure 6B).

## 4. Discussion

To date, little is known about the dynamics of host cytokine production induced by *E. tarda* infection. In this study, we utilized an antibody array to examine the cytokine response during *E. tarda* infection in mice at different time points. We detected 96 cytokines, 37 of which were DECs that exhibited significantly different expressions at 6 hpi and 24 hpi. The DECs enriched in the top GO, and KEGG terms/pathways were associated with cell migration and response to chemokine, immunity, disease, and infection, indicating an extensive induction of immune response by *E. tarda*. Furthermore, ten hub DECs with high levels of protein-protein interaction were identified, including CCL5, CXCL9, CXCL1, CXCL5, CCL3, IL6, and TNF–α. The potential significance of these hub cytokines is discussed below.

Chemokines are a group of small molecules (8 to 12 kD) that induce chemotaxis in a variety of cells and are vital for the clearance of pathogens during immune surveillance [44]. In this study, CCL5 and CXCL1 were the most robustly upregulated chemokines, with a fold change of 60.54 and 57.71, respectively, at 6 hpi. As a pro-inflammatory chemokine, CCL5 is known to mediate the trafficking and homing of lymphoid cells such as monocytes and T-cells and also act on the other cells, including eosinophils, basophils, dendritic cells, natural killer cells, and mast cells [45,46]. A previous study showed that CCL5 expression increased over time during *Mycobacterium tuberculosis* infection, and CCL5–knockout mice localized fewer antigen-presenting cells (APCs) and chemokine receptor-positive T-cells to the lungs in the early stage of *M. tb* infection [47]. CCL5 also plays a key role in the immune response to viral infection [48,49,50]. It has been reported that plasma CCL5 was markedly elevated in COVID–19 patients [51]. CXCL1 is a secreted factor that functions as a neutrophil chemoattractant [52]. Neutrophil migration to the site of bacterial infection is a crucial step in host defense. In a murine model of intrapulmonary *Streptococcus pneumoniae* infection, CXCL1 was found to enhance neutrophil influx to control bacterial dissemination in the lungs, resulting in improved host survival [53]. Similarly, CXCL1–transgenic mice constitutively expressing lung CXCL1 showed elevated neutrophil recruitment and bacterial clearance in the lungs, as well as enhanced host survival after *Klebsiella pneumoniae* infection [54]. Given these reports, it is likely that during the *E. tarda* infection in our study, the dramatic increase of CCL5 and CXCL1 likely represents an immune mechanism of the host to combat the invading *E. tarda*. Other chemokines, including CXCL9, CXCL5, and CCL3, were also identified as the key DECs in our study. CXCL9 has been reported to possess antimicrobial activity against bacterial pathogens such as *Citrobacter rodentium* [55,56]. CXCL5 was expressed by lung epithelial cells in response to *S. pneumoniae* infection and involved in neutrophil recruitment during inflammation response [57,58,59]. CCL3 has been shown to play a role in macrophage phagocytosis of *K. pneumonia* and is associated with the recruitment of leukocytes [60,61,62]. In our study, *E. tarda* infection significantly upregulated the expression of CXCL9, CXCL5, and CCL3, which may promote neutrophil recruitment and phagocytosis, resulting in enhanced bacterial clearance.

IL6 is a multifunctional cytokine involved in the regulation of acute-phase reaction, inflammatory response, and the transition from innate to adaptive immunity [63,64]. In our study, IL6 was the top DEC with extensive protein-protein interaction and was upregulated by *E. tarda* both in vivo and in vitro in macrophages. Previous studies have demonstrated that *E. tarda* is able to survive and replicate in host phagocytes, including macrophages [23,42]. In this study, we found that IL6 knockdown markedly increased the replication of *E. tarda* in macrophages, whereas the presence of rIL6 had the opposite effect, suggesting a requirement of IL6 in cellular defense against *E. tarda* invasion. A similar effect of IL6 has been observed in previous reports, which showed that mice with IL6 deficiency exhibited more severe *Escherichia coli* infections [65], and inhibition of IL6 signaling increased the survival of intracellular *Brucella abortus* in macrophages and decreased the production of TNF–α [66]. In our study, we observed that inhibition of IL6 significantly reduced iNOS, IL27, and TNF–α expression as well as NO production during *E. tarda* infection. iNOS is the major form of the enzyme that can generate NO, an important antimicrobial effector [67,68]. IL27 has a fundamental role in the regulation of innate and adaptive immunity and can be induced by IL6 during respiratory virus infection [69,70]. The enhanced *E. tarda* infection in RAW264.7 cells with IL6 knockdown observed in our study is likely due to the impaired ability of the cells to exert IL6–mediated inflammatory response.

TNF–α is a key regulator of the host response to the microbial challenge by amplifying and coordinating pro-inflammatory signals [71,72]. Bacterial infection studies in mammalian models showed that TNF–α deficiency caused an increased bacterial burden in *L. pneumophila* infected mice [73], and inhibition of TNF–α reduced NO and ROS production and promoted the infection of *Mycobacterium tuberculosis* and *Brucella abortus* [71,74,75]. In fish, zebrafish TNF–α was involved in NF–kB expression [76]; Nile tilapia TNF–α responded to *Streptococcus agalactiae* infection and participated in apoptosis [77]; the recombinant protein of grass carp TNF–α could induce the phosphorylation of IkBα [78]. In our study, TNF–α expression was found to be upregulated in *E. tarda*-infected mice, and rTNF–α treatment inhibited the intracellular replication of *E. tarda* in mouse macrophages, indicating an important role of TNF–α in the protective immunity against *E. tarda*. Since *E. tarda* is also a fish pathogen, we examined whether the findings in mice could be related to fish. Currently, no fish cell lines derived from macrophages or other phagocytes are available. In our study, we used the epithelial cells of Japanese flounder gill (FG–9307), which, like some mammalian epithelial cells, are known to be susceptible to *E. tarda* and have been used as a cellular model to study *E. tarda* infection [28,79,80]. We found that, similar to the observation in mice, TNF–α expression in flounder cells was upregulated by *E. tarda*, and that TNF–α knockdown significantly strengthened *E. tarda* infection in flounder cells, indicating that like mice, flounder also employed TNF–α in the regulation of immune defense against *E. tarda*. These results suggest a possible correlation between the immune responses of mice and fish caused by *E. tarda* infection.

In conclusion, in this study, we delineated the time–dependent plasma cytokine profile of mice during *E. tarda* infection. We identified 37 differentially expressed cytokines, including chemokines, interleukins, growth factors, and TNF superfamily members. Ten hub cytokines were further identified, which form complex interaction networks, and several of the hub cytokines were shown to be essential to effective *E. tarda* elimination. Our results add new insights into the importance of cytokines in mammal and fish immunity associated with bacterial infection and will serve as a foundation for future research on *E. tarda* infection and cytokine–mediated host immune defense.

## Figures and Tables

**Table 1:** Fold change (infected/uninfected group) of the DECs at 6 and 24 h post-infection (hpi).

DEC	Description	Fold Change	
		6 hpi	24 hpi
CCL5	Chemokine (C–C Motif) ligand 5	60.54	15.23
CXCL1	Chemokine (C–X–C Motif) ligand 1	57.71	8.93
IL6	Interleukin 6	35.08	2.34
CCL17	Chemokine (C–C Motif) ligand 17	33.78	2.87
CSF3	Colony-stimulating factor 3	29.23	30.23
CXCL9	Chemokine (C–X–C Motif) ligand 9	20.98	10.69
CCL20	Chemokine (C–C Motif) ligand 20	18.45	11.72
CXCL13	Chemokine (C–X–C Motif) ligand 13	11.76	28.47
TYRO3	TYRO3 protein tyrosine kinase 3	10.02	5.66
CCL12	Chemokine (C–C Motif) ligand 12	9.27	5.81
sTNF RII	Tumor necrosis factor receptor superfamily,	8.35	2.17
	member 1b		
IL12B	Interleukin 12b	7.84	0.64
CCL3	Chemokine (C–C Motif) ligand 3	6.73	1.35
CCL22	Chemokine (C–C Motif) ligand 22	6.39	7.69
CXCL5	Chemokine (C–X–C Motif) ligand 5	5.7	1.92
CX3CL1	Chemokine (C–X3–C Motif) ligand 1	4.74	1.17
CCL19	Chemokine (C–C Motif) ligand 19	4.22	1.46
MMP9	Matrix metallopeptidase 9	3.85	1.33
CSF1	Colony-stimulating factor 1	2.48	1.52
IFNγ	Interferon gamma	2.46	1.02
CXCL16	Chemokine (C–X–C Motif) ligand 16	2.45	2.47
VEGFA	Vascular endothelial growth factor-A	2.34	1.44
IL17A	Interleukin 17A	2.32	1.3
FGF2	Fibroblast growth factor 2	2.06	1.09
TIMP1	Tissue inhibitor of metalloproteinase 1	2.06	2.38
IL1α	Interleukin 1 alpha	1.97	2.13
sTNF RI	Tumor necrosis factor receptor superfamily,	1.95	1.37
	member 1a		
TNF-α	Tumor necrosis factor	1.92	1.69
CCL11	Chemokine (C–C Motif) ligand 11	1.85	2.64
CD40	CD40 Antigen	1.68	2.19
IGF-I	Insulin-like growth factor 1	0.53	0.06
VEGFD	Vascular endothelial growth factor D	1.2	9.95
IL17RB	Interleukin 17 receptor B	1.38	4.45
TNFRSF11B	Tumor necrosis factor receptor superfamily,	1.29	4.16
	Member 11b		
LEPR	Leptin receptor	1.21	3.63
MET	Met proto-oncogene	1.5	2.86
Flt3L	FMS–like tyrosine kinase 3 ligand	1.28	0.42

**Table 2:** Summary of the top 10 key differentially expressed cytokines (DECs) based on protein-protein interaction analysis.

DEC	Description	Number of Protein—Protein Interaction
IL6	Interleukin 6	26
TNF-α	Tumor necrosis factor	24
CCL5	Chemokine (C–C motif) ligand 5	19
CXCL9	Chemokine (C–X–C motif) ligand 9	19
IL17A	Interleukin 17A	17
CXCL1	Chemokine (C–X–C Motif) ligand 1	16
CXCL5	Chemokine (C–X–C Motif) ligand 5	15
CCL3	Chemokine (C–C Motif) ligand 3	14
CSF3	Colony stimulating factor 3	13
VEGFA	Vascular endothelial growth factor-A	13

## Data Availability

The data presented in this study are available in the article or Supplementary Materials.

## Supplementary Materials

The following are available online at https://www.mdpi.com/article/10.3390/biom11081242/s1, Table S1. The sequences of the siRNAs used for gene knockdown; Table S2. List of primers used for qRT–PCR; Figure S1. Verification of mouse IL6 (A) and Japanese flounder PoTNF–α (B) knockdown; Figure S2. *Edwardsiella tarda* dissemination in mouse tissues; Figure S3. Expression of IL6 *in Edwardsiella tarda*–infected RAW264.7 cells.
