# Supplementary material for: Cytokines Induced by Edwardsiella tarda: Profile and Role in Antibacterial Immunity
Source: Biomolecules. 2021 Aug 19;11(8):1242. doi: 10.3390/biom11081242 (PMC8391551; doi:10.3390/biom11081242)
Supplement: Supplementary file 1 [file biomolecules-11-01242-s001.zip › biomolecules-1285738-supplementary.pdf]

Supplementary data

Table S1. The sequences of the siRNAs used for gene knockdown.

siRNA	Forward primer (5′-3′)	Reverse primer (5′-3′)	Target gene
IL6si	GGAUGCUACCAAACUGGAUTT	AUCCAGUUUGGUAGCAUCCTT	Mouse IL6
PoTNF-αsi	GGCCAUCCAUUUAGAAGGUTT	ACCUUCUAAAUGGAUGGCCTT	Japanese flounder PoTNF-α
NCsi	UUCUCCGAACGUGUCACGUTT	ACGUGACACGUUCGGAGAATT	None

Table S2. List of primers used for qRT-PCR.

Gene name	Organism	Forward primer (5′-3′)	Reverse primer (5′-3′)
IL27	*Mus musculus*	CTTCCCAATGTTTCCCTGAC	CGAAGTGTGGTAGCGAGGA
IL6	*Mus musculus*	GGGAAATCGTGGAAATGAGA	AGGACTCTGGCTTTGTCTTTC
iNOS	*Mus musculus*	GAGCAACTACTGCTGGTGGT	CGATGTCATGAGCAAAGGCG
TNF-α	*Mus musculus*	GGACTAGCCAGGAGGGAGAA	CGCGGATCATGCTTTCTGTG
IL10	*Mus musculus*	TGGCCCAGAAATCAAGGAGC	CAGCAGACTCAATACACACT
GAPDH	*Mus musculus*	ATTCAACGGCACAGTCAAGG	GATGTTAGTGGGGTCTCGCTC
PoTNF-α	*Paralichthys olivaceus*	TACAGCCAGGCGTCATTCAG	GCCCAGGTAGATGGCATTGTA
Poβ-actin	*Paralichthys olivaceus*	AACCGCTGCCTCCTCCTCAT	TCGGGACAACGGAACCTCTC

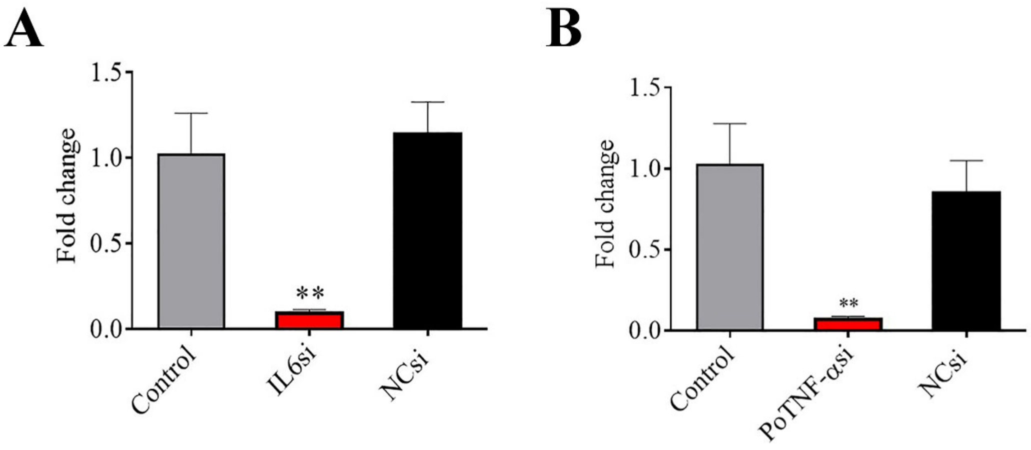

Figure S1. Verification of mouse IL6 (**A**) and Japanese flounder PoTNF-α **(B)** knockdown**.** (A) RAW264.7 cells were treated with or without (control) IL6si (a siRNA targeting IL6) or NCsi (negative control siRNA) for 24 h, and IL6 expression was determined by qRT-PCR. (B) Flounder FG-9307 cells were treated with or without (control) PoTNF-αsi (a siRNA targeting PoTNF-α) or NCsi for 24 h, and PoTNF-α expression was determined by qRT-PCR. Values are the means of triplicate experiments and shown as means ± SD. $^{**}p < 0.01$.

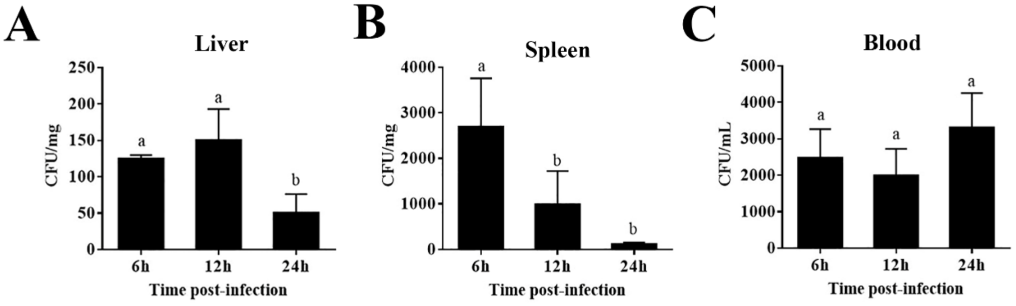

Figure S2. *Edwardsiella tarda* dissemination in mouse tissues. Mice were infected with *E. tarda* for different hours, and the bacterial numbers (Colony Forming Unit, CFU) in liver (A), spleen (B) and blood (C) were determined. Values are the means of three animals and shown as means ± SD. Different letters indicate statistical significance.

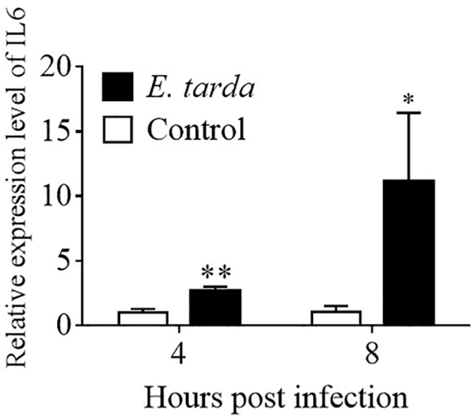

Figure S3. Expression of IL6 in *Edwardsiella tarda*-infected RAW264.7 cells. RAW264.7 cells were infected with or without (control) *E. tarda* for 1 h, and the extracellular bacteria were killed by antibiotic treatment. The cells were then incubated for 4 and 8 h, and IL6 expression was determined by qRT-PCR. Values are the means of triplicate experiments and shown as means ± SD. *$p < 0.05$; **$p < 0.01$.
